# Clinical outcomes and prognostic factors of Ommaya reservoir-based intrathecal chemotherapy in lung cancer leptomeningeal metastasis: a retrospective cohort study

**DOI:** 10.3389/fonc.2026.1915711

**Published:** 2026-09-28

**Authors:** Yuechao Yang, Mingtao Feng, Yu Xuan Ong, Zhe Qi, Bihani Pokharel, Lushao Zhang, Kexuan Wang, Jiatong Chen, Changshuai Zhou, Lei Chen, Deheng Li, Zhihuang Hu, Liangdong Li, Yiqun Cao, Yang Gao

**Affiliations:** 1 Department of Neurosurgery, Fudan University Shanghai Cancer Center, Shanghai, China; 2 Department of Oncology, Shanghai Medical College, Fudan University, Shanghai, China; 3 Shanghai Medical College, Fudan University, Shanghai, China; 4 Department of Medical Oncology, Fudan University Shanghai Cancer Center, Shanghai, China

**Keywords:** intrathecal chemotherapy, leptomeningeal metastasis, lung cancer, ommaya reservoir, prognostic factors

## Abstract

**Objectives:**

To evaluate clinical outcomes and identify prognostic determinants in patients with lung cancer leptomeningeal metastasis (LCLM) treated with Ommaya reservoir-based intrathecal (IT) chemotherapy.

**Method:**

We retrospectively reviewed 154 LCLM patients treated at Fudan University Shanghai Cancer Center (2022-2025). All patients underwent Ommaya reservoir implantation, or combined with VP shunt. IT pemetrexed (30 mg/week) was delivered for 4–6 consecutive weeks. Clinical characteristics, cerebrospinal fluid (CSF) and magnetic resonance imaging (MRI) features were collected. Survival outcomes were assessed using Kaplan–Meier and Cox regression analyses.

**Results:**

The cohort had a median age of 59 years (IQR, 53-65) with predominance of female sex (59.1%), lung adenocarcinoma (98.1%), and EGFR mutations (76.7%). Median overall survival (OS) for the entire cohort was 331 days (IQR, 266–446). To provide guidance on clinical decision making, we established an exploratory four-tier prognostic system integrating KPS and intracranial pressure (ICP), categorizing patients into distinct prognostic quartiles. Our analysis revealed that among patients with Karnofsky Performance Status (KPS) ≥70, a normal ICP was associated with superior survival. Conversely, in patients with KPS ≤ 50, a decrease in intracranial pressure indicates a poorer prognosis for the patients. Multivariate Cox regression identified KPS, extracranial metastasis, MRI morphology, and CSF glucose/protein as independent prognostic factors. A nomogram developed from these variables showed excellent discrimination (AUC = 0.894) and robust calibration.

**Conclusions:**

This study establishes a practical risk stratification system and a prognostic nomogram to assist individualized clinical decision-making for LCLM patients.

## 1 Introduction

Lung cancer leptomeningeal metastasis (LCLM) is a severe complication of advanced-stage lung cancer progression with a median survival time of only a few weeks to months (1, 2). Its incidence is approximately 3.8%, occurring most often in adenocarcinoma, particularly in patients with epidermal growth factor receptor (EGFR) mutations or anaplastic lymphoma kinase (ALK) rearrangements (3). The rising incidence reflects improved diagnostic sensitivity and prolonged systemic disease control, allowing central nervous system (CNS) progression to emerge (4, 5).

Due to the unique structure of the leptomeninges and the presence of the blood-brain barrier (BBB), LM often presents with various neurological symptoms such as seizure, headache, nausea, and cranial nerve palsy. LM is believed to arise mainly through hematogenous dissemination at the choroid plexus epithelial cells, after which tumor cells circulate in the cerebrospinal fluid (CSF) and adhere to or float along the meningeal surfaces (6, 7). These adherent and floating phenotypes can interconvert, contributing to heterogeneity in drug response and resistance (8). The goals of LM treatment include alleviating neurological symptoms, improving quality of life, and prolonging survival (9).

Traditional systemic chemotherapy and molecular targeted drugs could not effectively penetrate the BBB, resulting in subtherapeutic concentrations in the CNS and markedly limited therapeutic efficacy. Intraventricular intrathecal (IT) chemotherapy, a local administration method, bypasses the BBB by direct injection into the cerebrospinal fluid and achieves high drug concentrations along the leptomeninges surface, offering a promising strategy for LM control (10–12). However, IT via repeated lumbar puncture (LP) is constrained by frequent invasive procedures, local chemical arachnoiditis, patient discomfort, and poor long-term compliance (13). To facilitate repeated IT administration, the Ommaya reservoir has become a widely adopted device for long-term intraventricular access. In patients with concurrent hydrocephalus, Ommaya reservoir is combined with a ventriculoperitoneal (VP) shunt to simultaneously manage CSF flow and deliver therapy, providing dual therapeutic advantage.

Despite its widespread clinical use, the clinical efficacy and prognostic determinants of IT therapy remain controversial (14, 15). Many existing studies suffer from small sample sizes, single-center retrospective designs, and incomplete integration of clinical, biochemical, and imaging parameters, limiting the ability to accurately identify patients who may benefit most from IT treatment. In addition, LM diagnosis remains challenging. Although neurological symptoms, MRI, and CSF cytology constitute the current diagnostic framework (9), their limited sensitivity and specificity, marked heterogeneity in clinical manifestations, imaging patterns, and CSF biomarkers complicate disease classification and prognosis stratification (6).

A range of prognostic factors have been reported, including ECOG, KPS (16), age (17), CSF biomarkers (18–20), molecular alterations (21, 22), neutrophil-to-lymphocyte ratio (NLR) (23), and neurological symptoms (24–26), while emerging biomarkers such as ctDNA (27) and circulating tumor cells (CTCs) (28) offer additional promise. Despite this, prognostic results vary widely, reflecting heterogeneity in patient populations, diagnostic criteria, and treatment eras. These limitations highlight the need for an integrated model combining clinical, biochemical, and imaging variables for more accurate risk stratification.

Previous studies on Ommaya-based IT chemotherapy for LCLM were limited by small sample sizes (15). We retrospectively analyzed 154 patients with LCLM treated with IT pemetrexed delivered via an Ommaya reservoir, either alone or combined with a VP shunt. We systematically evaluated the impact of IT therapy on survival, investigated the prognostic value of demographic characteristics, disease burden, CSF biochemical indices, and MRI enhancement patterns. In addition, a novel four-tier prognostic classification system was established for stratification of patients into subgroups based on Karnofsky performance status (KPS) and intracranial pressure (ICP) to assist clinical risk assessment. Furthermore, we developed a nomogram based on independent factors to support risk stratification and personalized treatment planning in clinical practice.

## 2 Materials and methods

### 2.1 Study population

This retrospective study included 154 patients diagnosed with LCLM who were treated at the Department of Neurosurgery, Fudan University Shanghai Cancer Center, between January 2022 and July 2025.

Inclusion criteria were as follows:

1. a confirmed diagnosis of lung cancer with radiologically or cytologically proven LM;
2. available pre-treatment MRI and CSF analysis;
3. complete clinicopathologic and follow-up data.

All patients had previously received standard systemic therapy including chemotherapy and/or targeted agents and subsequently developed resistance.

Exclusion criteria included:

1. primary CNS tumors;
2. severe systemic disease precluding follow-up;
3. missing key imaging or biochemical data.

Clinical and pathological data were collected from electronic medical records, including age, gender, histological subtype, EGFR or ALK mutation status, KPS, ICP, MRI characteristics, CSF biochemical results (protein, glucose, chloride, LDH), and treatment modalities. KPS was used to evaluate neurological and functional performance, with scores ranging from 0 to 100; higher scores indicated better physical function. This retrospective study included 154 patients diagnosed with LCLM who were treated at the Department of Neurosurgery, Fudan University Shanghai Cancer Center, between January 2022 and July 2025. The enrollment period spanned from July 1, 2025, to November 30, 2025. The minimum follow-up duration for all participants was 4 months. Median follow-up time was calculated using the reverse Kaplan-Meier method, yielding a value of 338 days for the overall cohort. The overall censoring rate was 59.0%. Written informed consent for the use of clinical data for research purposes was obtained from all participants or their legal guardians, in accordance with the requirement of the Institutional Ethics Committee.

### 2.2 Neuroimaging evaluation

All patients underwent contrast-enhanced MRI of the brain before treatment. Imaging features were reviewed independently by two experienced neuroradiologists blinded to clinical outcomes.

MRI enhancement patterns were classified as linear, nodular, or mixed based on meningeal enhancement distribution. Patients with both linear and nodular enhancement were categorized as the mixed pattern. Ventricular enlargement and periventricular edema were also assessed. Ventricular enlargement was defined based on pre-treatment MRI at admission, with ventricular dimensions exceeding the upper limit of the normal reference range for age-adjusted adults. Extracranial metastases were confirmed by PET-CT, and the presence of bony spinal metastases was assessed by spinal MRI or PET-CT, defined as focal osteolytic or osteosclerotic lesions in the vertebrae or adjacent structures.

### 2.3 CSF analysis and ICP measurement

LP was performed in all patients before the initiation of intracranial treatment. Opening pressure was recorded in millimeters of water (mmH_2_O), and intracranial hypertension was defined as an opening pressure ≥ 200 mmH_2_O. CSF samples were collected simultaneously for biochemical and cytological analyses. Protein, glucose, chloride, and LDH levels were measured using standardized automated biochemical analyzers. Cytological assessment for malignant cells was performed by experienced cytopathologists. Associations between ICP and MRI features (nodular or linear enhancement, ventricular enlargement, and periventricular edema) were further analyzed.

### 2.4 Treatment and follow-up

As one of the earliest centers to standardize IT chemotherapy for LCLM, all 154 patients underwent Ommaya reservoir implantation for IT drug delivery under general anesthesia. After recovery from surgery, patients received standard IT chemotherapy with pemetrexed (30 mg) administered through the Ommaya reservoir once every 1–2 weeks, according to institutional protocols.

If necessary, during the Ommaya implantation procedure, patients with symptoms of hydrocephalus or persistently elevated ICP underwent a VP shunt procedure. Indications for VP shunt placement included: (1) opening pressure ≥ 200 mmH_2_O with symptomatic hydrocephalus; and (2) in borderline cases (ICP 180–200 mmH_2_O), a positive CSF tap test (defined as clinical improvement within 24–48 hours after lumbar drainage) was required prior to shunt placement. All decisions were made by a multidisciplinary team. All VP shunts placed were programmable devices. During IT chemotherapy infusion via the Ommaya reservoir, the shunt was not clamped; instead, slow infusion (over 5–10 minutes) was performed with close monitoring for clinical signs of overdrainage. Shunt settings were adjusted postoperatively based on clinical and radiological follow-up. Systemic treatments, including targeted therapy (e.g., EGFR-TKIs or ALK inhibitors) and radiotherapy, were provided based on molecular and clinical characteristics.

Patients were followed every 3 months with neurological assessments and contrast-enhanced MRI. CSF cytology and biochemical parameters were re-examined periodically to evaluate treatment response and disease progression. Overall survival (OS) was defined as the time from LM diagnosis to death or last follow-up, and patients alive at the last contact were censored. Follow-up continued until July 31, 2025.

### 2.5 Statistical analysis

All statistical analyses were performed using R software (version 4.3.1; R Foundation for Statistical Computing, Vienna, Austria). Continuous variables were expressed as median (interquartile range, IQR), and categorical variables as counts and percentages. Comparisons between groups were performed using the Mann–Whitney U test or Fisher’s exact test, as appropriate. Survival curves were generated using the Kaplan–Meier method, and group differences were compared using the log-rank test. Univariate and multivariate Cox proportional hazards models were applied to identify independent prognostic factors for OS. Variables with p < 0.05 in univariate analysis were included in the multivariate model. A prognostic nomogram for 1-year survival was constructed using the *rms* package, based on significant predictors identified in multivariate analysis. Internal validation of the Cox nomogram was performed via 1000-times bootstrap resampling to quantify overfitting optimism. The unadjusted apparent C-index and optimism-corrected C-index after resampling were both calculated. Calibration intercept and slope for 1-year survival prediction were derived from the bootstrap calibration curve, and time-dependent AUC at 365 days was computed to quantify discriminative capacity. Two-sided p*-*values < 0.05 were considered statistically significant.

## 3 Results

### 3.1 Patient characteristics

A total of 154 patients with LCLM were included in the analysis (**Table 1**). The median age was 59 years (IQR, 53–65), with a female predominance (59.1%, n = 91). Adenocarcinoma was the predominant histologic subtype (98.1%, n = 151), and EGFR mutations were identified in 76.6% (n = 118). VP shunts were placed in 58.4% of patients (n = 90). MRI revealed heterogeneous enhancement patterns, including nodular enhancement in 48.1% (n = 74), linear enhancement in 52.6% (n = 81), periventricular edema in 53.9% (n = 83), and ventricular enlargement in 48.7% (n = 75). The distribution of enhancement types was mixed (24.7%), nodular (23.4%), linear (27.9%), and absent (24.0%). Systemically, extracranial metastasis was observed in 55.8% (n = 86) of patients, while spinal involvement was less common (18.8%, n = 29). Prior resection of the primary lung tumor had been performed in 46.8% of patients (n = 72). CSF cytology confirmed malignant cells in 87.7% of patients (n = 135).

**Table 1:** Baseline characteristics of LCLM patients.

Variable	N = 154^1^
Gender	
Male	63.0 (40.9%)
Female	91.0 (59.1%)
Pathological type	
Adenocarcinoma	151.0 (98.1%)
Large cell neuroendocrine carcinoma	1.0 (0.6%)
Small cell lung cancer	2.0 (1.3%)
Gene mutation	
EGFR	118.0 (76.6%)
KRASp, NTRK2	1.0 (0.6%)
No mutation	35.0 (22.7%)
Ventriculoperitoneal shunt	
0	64.0 (41.6%)
1	90.0 (58.4%)
MR nodular enhancement	
0	80.0 (51.9%)
1	74.0 (48.1%)
MR linear enhancement	
0	73.0 (47.4%)
1	81.0 (52.6%)
Periventricular edema	
0	71.0 (46.1%)
1	83.0 (53.9%)
Ventricular enlargement	
0	79.0 (51.3%)
1	75.0 (48.7%)
Primary tumor resection	
0	82.0 (53.2%)
1	72.0 (46.8%)
Extra-cranial metastasis	
0	68.0 (44.2%)
1	86.0 (55.8%)
Spinal metastasis	
0	125.0 (81.2%)
1	29.0 (18.8%)
CSF cytology for tumor cells	
0	19.0 (12.3%)
1	135.0 (87.7%)
MRI enhancement pattern	
Mixed enhancement	38.0 (24.7%)
Nodular enhancement	36.0 (23.4%)
Linear enhancement	43.0 (27.9%)
None	37.0 (24.0%)
Chemotherapy-related complications	
Nausea and vomiting	56.0 (36.4%)
Epileptic seizure	10.0 (6.5%)
Bone marrow suppression	9.0 (5.8%)
None	79.0 (51.3%)
Surgical-related complications	
Subcutaneous effusion	5.0 (3.2%)
Wound infection	2.0 (1.3%)
Surgical site bleeding	1.0 (0.6%)
None	146.0 (94.8%)
**Age (years)**	59.0 (53.0, 65.0)
**KPS**	60.0 (50.0, 70.0)
**Lumbar puncture pressure (mmH_2_O)**	220.0 (160.0, 290.0)
**LDH (U/L)**	39.0 (21.0, 59.0)
**GLU (mmol/L)**	2.4 (1.4, 3.2)
**CL (mmol/L)**	122.0 (118.0, 125.0)
**Protein (mg/L)**	629.0 (453.0, 816.0)
Event	
0	91.0 (59.1%)
1	63.0 (40.9%)
**Overall Survival (days)**	331.0 (266.0, 446.0)

^1^ n (%); Median (Q1, Q3).

Bold values indicate statistical significance.

Baseline neurological function was impaired in most patients, with a median KPS score of 60 (IQR, 50–70). The median opening pressure during LP was 220 mmH_2_O (IQR, 160–290). CSF biochemical analysis showed median LDH 39 U/L (IQR, 21–59), glucose 2.4 mmol/L (IQR, 1.4–3.2), chloride 122 mmol/L (IQR, 118–125), and protein 629 mg/L (IQR, 453–816). Regarding treatment-related adverse events, chemotherapy administered via the Ommaya reservoir was generally well tolerated. The most common chemotherapy-related complications included nausea and vomiting (36.4%, n = 56), epileptic seizures (6.5%, n = 10), and bone marrow suppression (5.8%, n = 9), while 51.3% (n = 79) reported none. Surgical complications following VP shunt placement were rare, including subcutaneous effusion (3.2%, n = 5), wound infection (1.3%, n = 2), and surgical site bleeding (0.6%, n = 1); 94.8% (n = 146) of patients had no surgical complications. During follow-up, 63 patients (40.9%) experienced death or disease progression. The median OS was 331 days (IQR, 266–446). A comparative baseline analysis between patients receiving VP shunts and those without is summarized in **Supplementary Table S1**. Patients who underwent shunt placement presented significantly younger age, higher intracranial pressure and more frequent periventricular edema consistent with shunt indications, whereas overall survival did not differ significantly between the two subgroups (*P* = 0.680).

### 3.2 Prognostic impact of clinical parameters

The prognostic value of clinical parameters was evaluated using Kaplan–Meier survival analysis with log-rank testing. There were no significant differences in OS between age groups (HR = 1.042, *p* = 0.734) (**Figure 1a**). Female patients survived significantly longer than males (HR = 1.661, *p* < 0.05) (**Figure 1b**). Higher KPS was strongly associated with prolonged survival (HR = 0.551, *p* < 0.05) (**Figure 1c**). Patients with normal CSF glucose levels (≥ 2.6 mmol/L) demonstrated better outcomes (HR = 0.423, *p* < 0.005) (**Figure 1d**), whereas elevated CSF protein (≥ 450 mg/L) predicted poorer prognosis (HR = 2.947, *p* < 0.005) (**Figure 1e**). In contrast, CSF LDH levels were not significantly correlated with survival (HR = 0.729, *p* = 0.151) (**Figure 1f**).

**Figure 1:**
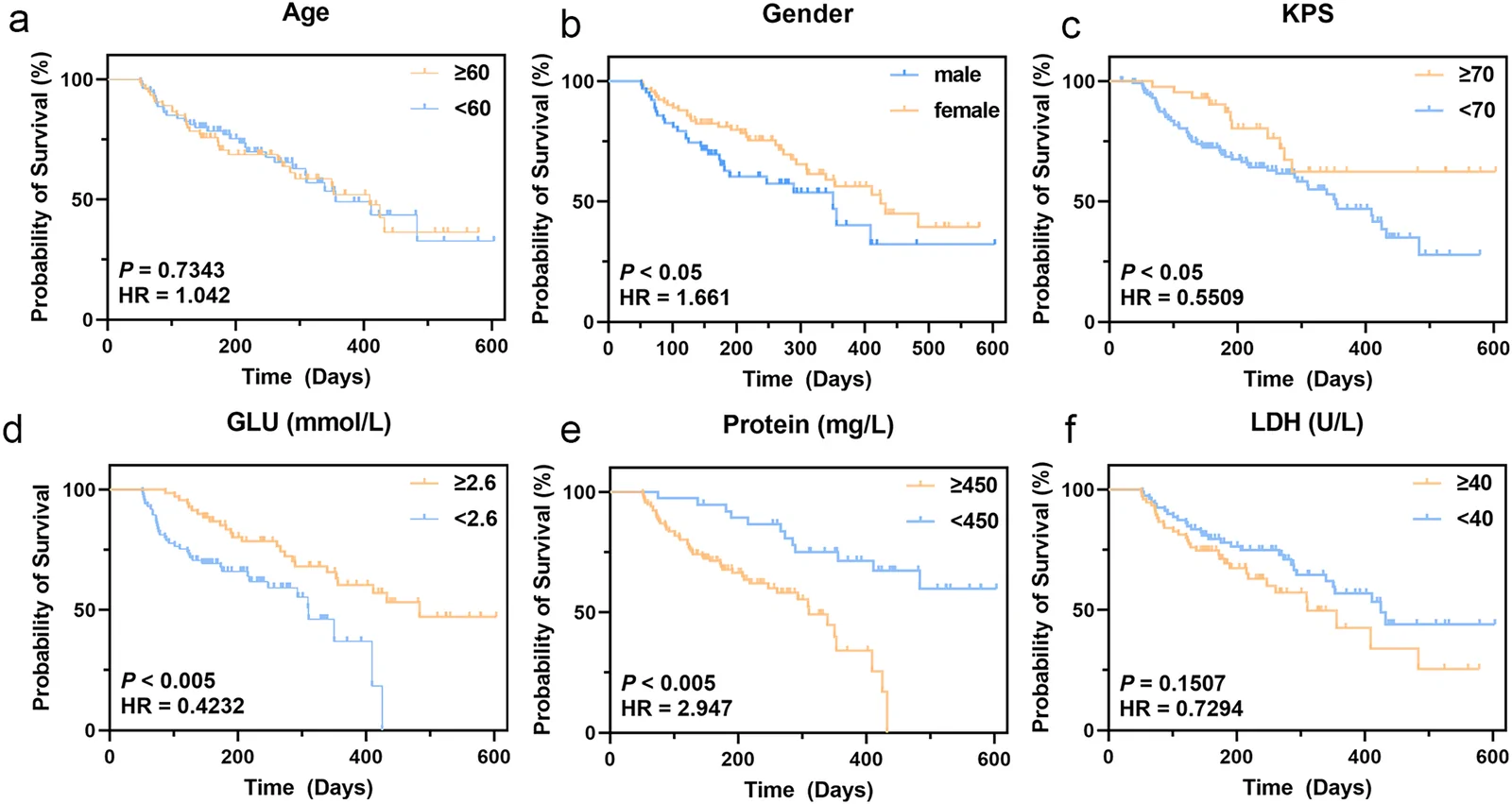
Kaplan-Meier survival analysis of the association between baseline clinical parameters and OS in LCLM patients. **(a)** No association was observed between age groups and OS (HR = 1.042, *p* = 0.734). **(b)** Female patients had significantly longer OS compared to male patients (HR = 1.661, *p* < 0.05). **(c)** A higher KPS score was associated with significantly prolonged OS (HR = 0.551, *p* < 0.05). **(d)** Patients with elevated CSF glucose levels demonstrated better OS (HR = 0.423, *p* < 0.005). **(e)** Patients with high CSF protein concentrations (≥ 450 mg/L) had a markedly worse prognosis (HR = 2.947, *p* < 0.005). **(f)** CSF LDH levels were not significantly correlated with OS (HR = 0.729, *p* = 0.151). (n=154 for all analyses).

LP-measured ICP did not affect overall survival (HR = 0.995; *p* = 0.810; **Figure 2a**). However, subgroup analysis revealed that elevated ICP predicted poorer survival among patients with KPS ≥ 70 (HR = 3.401; *p* < 0.05; **Figure 2b**), whereas no association was observed in those with KPS ≤ 50 (**Figure 2c**).

**Figure 2:**
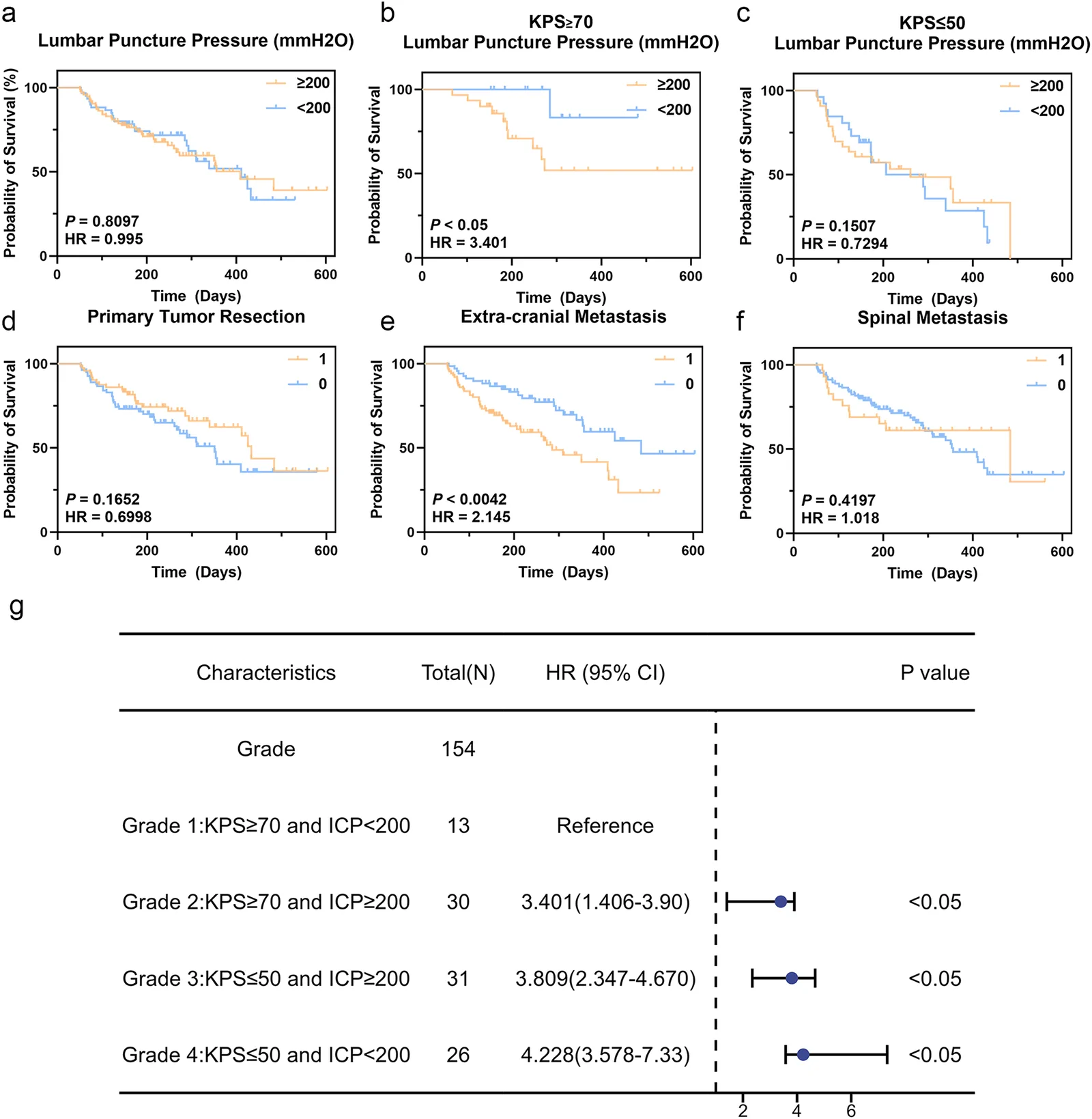
Kaplan-Meier survival analysis of the impact of ICP, primary tumor resection, and metastasis on OS. **(a)** LP-measured ICP was not significantly related to OS (HR = 0.995, *p* = 0.810). (b, c) Subgroup analysis based on KPS showed that elevated ICP was associated with poorer survival in patients with KPS ≥ 70 (b; HR = 3.401; *p* < 0.05), but not in those with KPS ≤ 50 **(c)**. **(d)** Primary lung tumor resection did not confer a significant survival advantage (HR = 0.6998, *p* = 0.1652). **(e)** The presence of extracranial metastasis was significantly associated with worse prognosis (HR = 2.145, *p* < 0.004). **(f)** Spinal metastasis had no significant effect on OS (HR = 1.018, *p* = 0.4197). (n=154 for overall analyses). **(g)** The grading is based on the assessment of prognosis using KPS and ICP.

Primary lung tumor resection did not confer a survival advantage (HR = 0.700; *p* = 0.165; **Figure 2d**). In contrast, extracranial metastasis was significantly linked to worse prognosis (HR = 2.145; *p* = 0.004; **Figure 2e**), while spinal metastasis had no significant effect on survival (HR = 1.012; *p* = 0.420; **Figure 2f**). We categorized patients into four prognostic grades based on KPS and intracranial pressure: Grade 1 (KPS ≥ 70, ICP < 200 mmH_2_O), Grade 2 (KPS ≥ 70, ICP ≥ 200 mmH_2_O), Grade 3 (KPS ≤ 50, ICP ≥ 200 mmH_2_O), Grade 4 (KPS ≤ 50, ICP < 200 mmH_2_O). The hazard ratio rose stepwise from Grade 1 to Grade 4 (**Figure 2g**), with each higher grade associated with significantly greater mortality risk relative to Grade 1 (all *P* < 0.05; full survival data in **Supplementary Table S2**). No statistically significant survival differences were observed in Bonferroni-adjusted pairwise comparisons among Grades 2-4 (**Supplementary Table S3**), likely due to limited subgroup sample sizes and overlapping baseline disease severity, which reduced statistical power to detect minor prognostic gaps between these three high-risk strata. Further large-sample cohort analyses are required to validate the survival distinctions between these intermediate risk grades. Grade 1 only included 13 patients with a single death event, and its median overall survival was estimated as not reached. This survival estimate carries low statistical robustness due to the extremely small sample size and limited endpoint events, and clinicians should avoid overinterpreting the long-term survival advantage of this subgroup.

### 3.3 Radiographic features and survival associations

Distinct MRI enhancement patterns exhibited clear prognostic implications. Patients with nodular enhancement demonstrated significantly shorter OS (HR = 2.947; *p* < 0.0005; **Figure 3a**), reflecting the aggressive biological behavior of focal meningeal infiltration. In contrast, linear enhancement conferred prolonged survival (HR = 0.618; *p* < 0.05; **Figure 3b**), suggesting that diffuse leptomeningeal involvement may respond better to IT chemotherapy. Other radiographic features showed limited prognostic relevance. Ventricular enlargement (HR = 1.129; *p* = 0.707; **Figure 3c**) and periventricular edema (HR = 1.339; *p* = 0.311; **Figure 3d**) were not significantly associated with OS, suggesting that these findings may reflect secondary CSF flow obstruction rather than direct tumor activity. Patients with nodular or mixed enhancement had significantly poorer survival than those with linear enhancement (*p* < 0.001; **Figure 3e**), confirming the adverse prognosis associated with nodular lesions.

**Figure 3:**
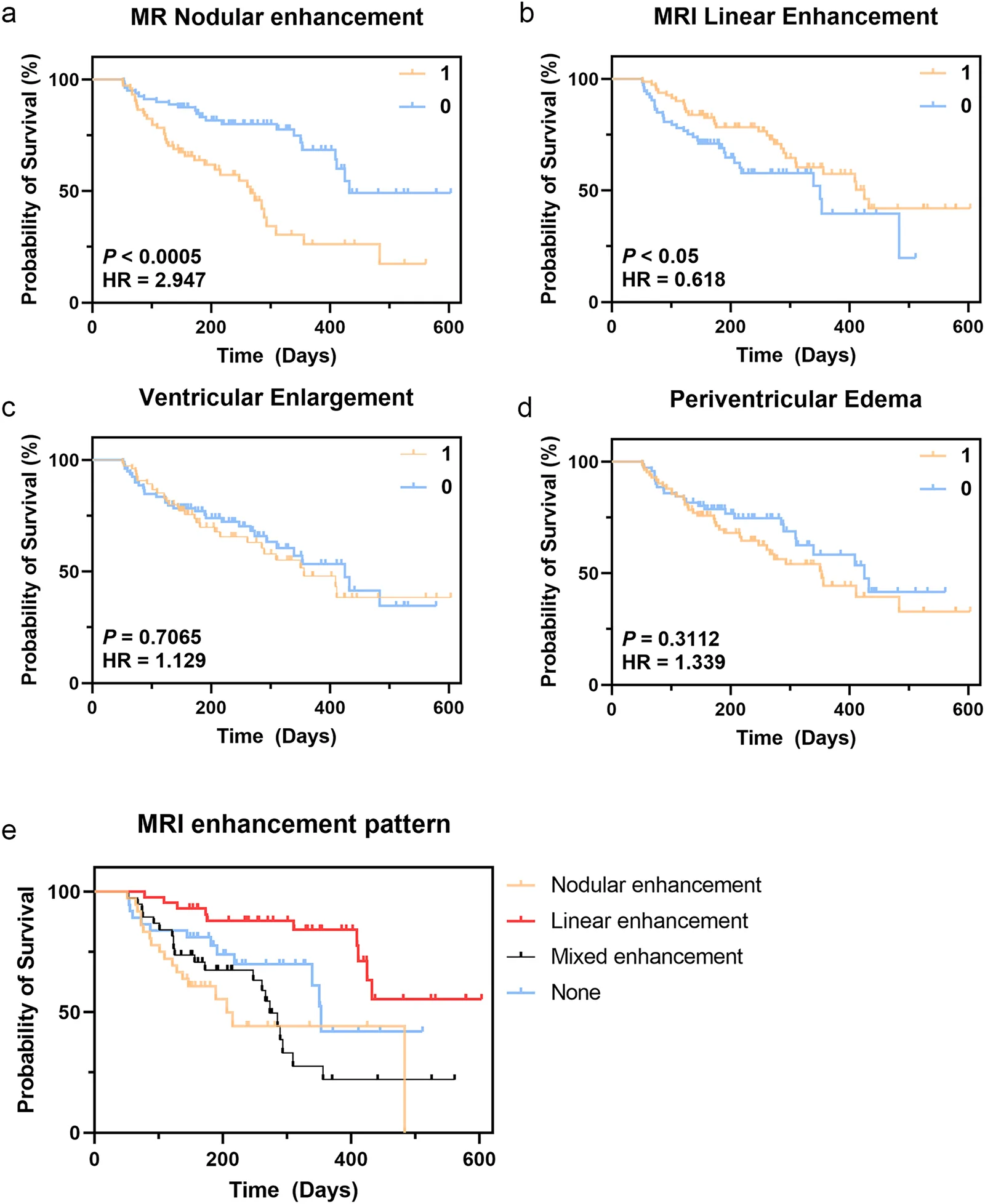
Prognostic implications of MRI enhancement patterns on OS. **(a)** Patients with nodular enhancement had significantly shorter OS compared to other patterns (HR = 2.947, *p* < 0.0005). **(b)** Linear enhancement was associated with prolonged survival (HR = 0.618, *p* < 0.05). **(c)** Ventricular enlargement was not significantly associated with OS (HR = 1.129, *p* = 0.707). **(d)** Periventricular edema was not significantly associated with OS (HR = 1.339, *p* = 0.311). **(e)** Comprehensive analysis of enhancement patterns showed that patients with nodular or mixed enhancement had significantly poorer survival than those with linear enhancement (*p* < 0.05). (n=154 for all analyses).

Enhanced MRI demonstrated the typical pattern of meningeal metastasis enhancement (**Figure 4a**). CSF pressure measurements provided further insight into the radiologic–clinical relationship. Elevated ICP was observed in 61.0% of patients, while normal and decreased ICP were seen in 37.7% and 1.3%, respectively (**Figure 4b**). High ICP was positively correlated with nodular enhancement (*p* < 0.005; **Figure 4c**), suggesting that intracranial hypertension often reflects extensive and space-occupying meningeal involvement. Interestingly, patients with linear enhancement also tended to present with elevated ICP (*p* < 0.05), possibly due to diffuse leptomeningeal inflammation and CSF flow resistance. Additionally, periventricular edema showed a strong association with elevated ICP (*p* < 0.0005; **Figure 4e**), whereas ventricular enlargement demonstrated no significant correlation (**Figure 4f**), implying that edema formation may be a more sensitive imaging marker of intracranial hypertension than ventricular size.

**Figure 4:**
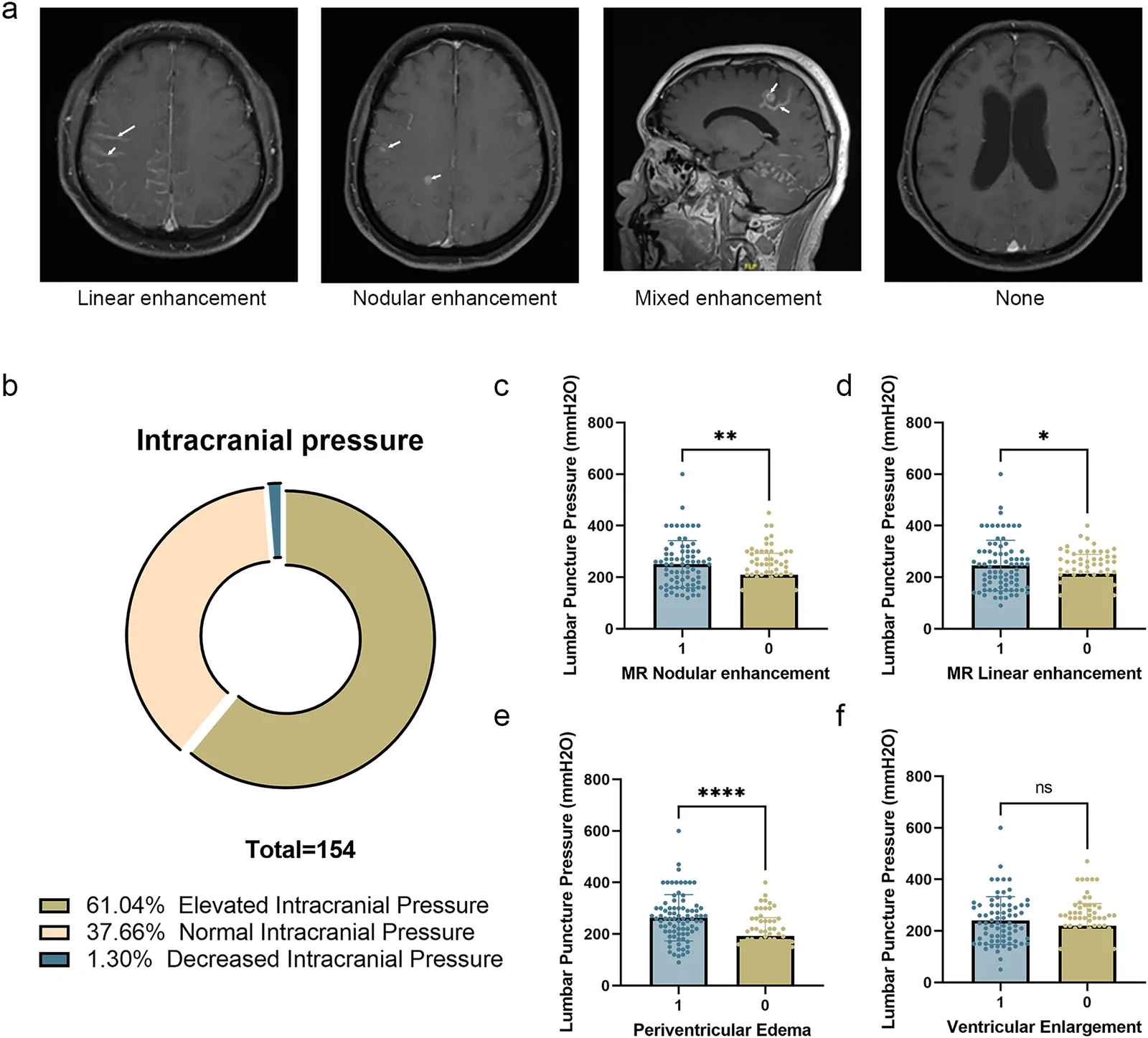
Radiographic features and their correlation with ICP. **(a)** Representative enhanced MRI showing a typical pattern of meningeal metastasis enhancement. **(b)** Pie chart showing the distribution of patients based on ICP levels (elevated: 61.0%, normal: 37.7%, decreased: 1.3%). **(c)** Elevated ICP was positively correlated with the presence of nodular enhancement (*p* < 0.005). **(d)** Elevated ICP was also associated with linear enhancement (*p* < 0.05). **(e)** Periventricular edema showed a strong association with elevated ICP (*p* < 0.0005). **(f)** Ventricular enlargement demonstrated no significant correlation with ICP. (n=154 for correlations). * p < 0.05, ** p < 0.01, **** p < 0.0001, ns > 0.05.

### 3.4 Functional outcome and post-treatment KPS changes

Neurological function improved in the majority of patients following treatment. Overall, 71.4% showed increased KPS, 16.9% remained stable, and 11.7% showed deterioration after combined Ommaya reservoir–based IT chemotherapy and systemic therapy (**Figure 5a**).

**Figure 5:**
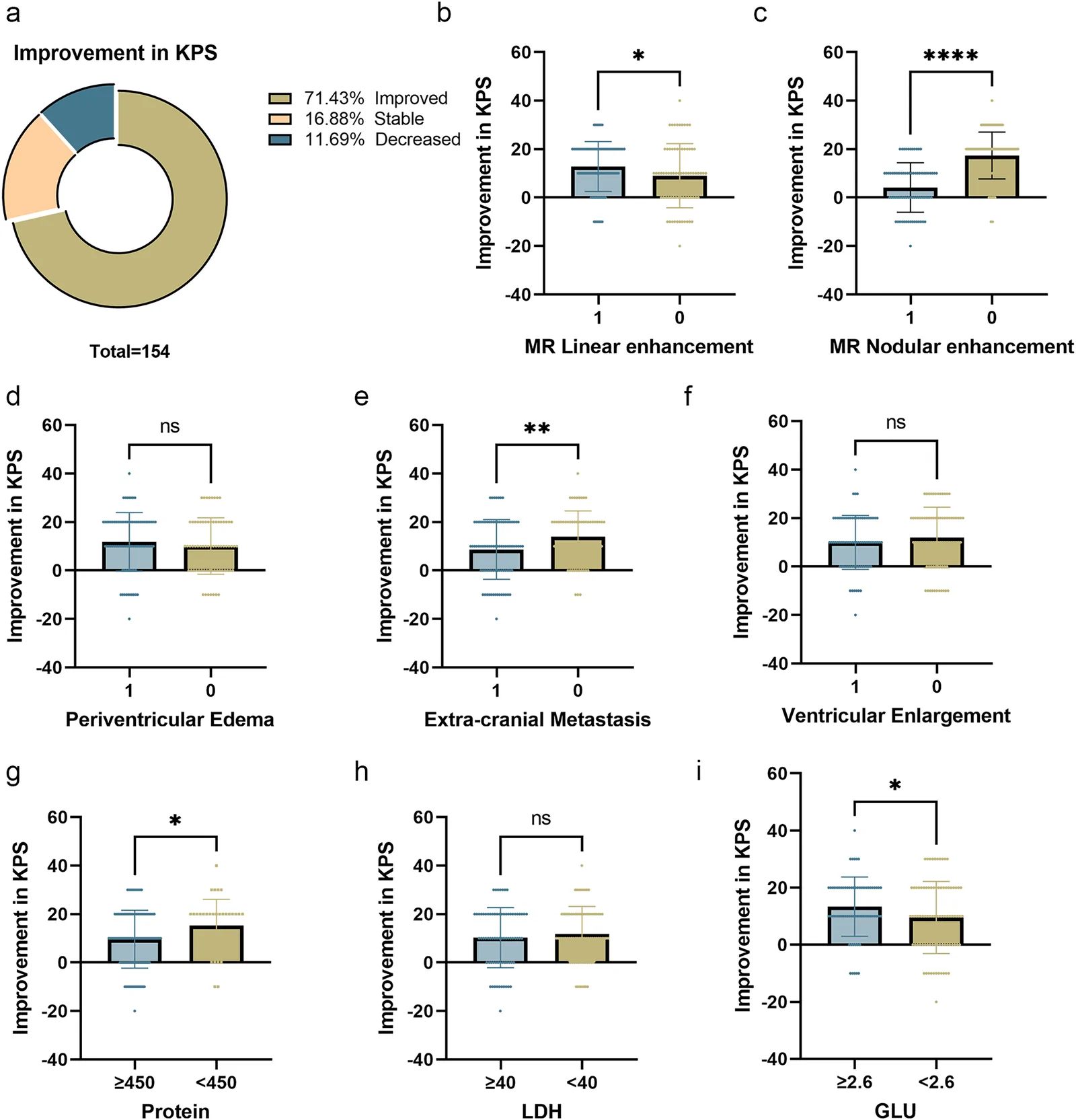
Post-treatment changes in KPS and associated factors. **(a)** Overall distribution of KPS changes after combined Ommaya reservoir-based IT chemotherapy and systemic therapy (improvement: 71.4%, stable: 16.9%, deterioration: 11.7%). **(b)** Patients with linear enhancement on baseline MRI demonstrated significantly higher rates of KPS improvement (*p* < 0.05). **(c)** Patients with nodular enhancement experienced markedly poorer functional recovery (*p* < 0.05). (d-f) The presence of periventricular edema **(d)** or ventricular enlargement **(e)** showed no significant association with KPS improvement (*p* > 0.05). **(f)** The presence of extracranial metastasis was associated with a significantly reduced likelihood of KPS improvement (*p* < 0.05). **(g)** Patients with elevated CSF protein (≥ 450 mg/L) had significantly poorer functional recovery (*p* < 0.05). **(h)** CSF LDH levels were not associated with KPS changes (*p* > 0.05). **(i)** Patients with higher CSF glucose (≥ 2.6 mmol/L) showed significantly greater improvement in KPS (*p* < 0.05). (n=154 for overall cohort, subgroup n values vary). * p < 0.05, ** p < 0.01, **** p < 0.0001, ns > 0.05.

Linear enhancement predicted the greatest neurological recovery, with higher KPS improvement than other patterns (*p* < 0.05; **Figure 5b**). In contrast, nodular enhancement predicted poorer functional recovery (*p* < 0.05; **Figure 5c**), consistent with its association with more aggressive LM and elevated ICP. Patients with periventricular edema (**Figure 5d**) or ventricular enlargement (**Figure 5f**) had limited neurological improvement; however, these differences were not statistically significant (*p* > 0.05), suggesting that these MRI features reflect secondary CSF circulation disturbances rather than direct determinants of neurological outcome. The presence of extracranial metastasis was associated with significantly reduced likelihood of post-treatment KPS improvement (*p* < 0.05; **Figure 5e**), indicating that systemic tumor burden negatively influenced recovery despite intracranial disease control.

Biochemical parameters further supported these findings. Patients with elevated CSF protein levels (≥ 450 mg/L) had significantly poorer post-treatment functional recovery compared with those with normal protein levels (HR = 2.947; *p* < 0.05; **Figure 5g**). In contrast, CSF LDH levels were not associated with KPS (*p* > 0.05; **Figure 5h**). Conversely, patients with normal CSF glucose (≥ 2.6 mmol/L) demonstrated significantly greater KPS improvement after IT chemotherapy (*p* < 0.05; **Figure 5i**), suggesting that preserved metabolic homeostasis in the CSF microenvironment may contribute to better neurological recovery.

### 3.5 Survival analysis and prognostic model development

Univariate and multivariate Cox regression analyses identified several prognostic factors associated with OS (**Table 2**). In univariate analysis, favorable predictors included female gender (HR = 0.546, *p* = 0.020), higher KPS (HR = 0.951, *p* < 0.001), absence of extracranial metastasis (HR = 0.458, *p* = 0.004), normal CSF glucose (HR = 0.506, *p* < 0.001), higher CSF chloride (HR = 0.936, *p* = 0.010), and absence of MR nodular enhancement (HR = 0.366, *p* < 0.001).

**Table 2:** Risk factors for LCLM.

Characteristics	Total(N)	Univariate analysis		Multivariate analysis	
		Hazard ratio (95% CI)	*P* value	Hazard ratio (95% CI)	*P* value
Gender	154				
Male	63	Reference		Reference	
Female	91	0.546 (0.329 - 0.909)	**0.020**	0.784 (0.444 - 1.382)	0.400
Age	154	0.983 (0.956 - 1.010)	0.204		
KPS	154	0.951 (0.931 - 0.971)	**< 0.001**	0.969 (0.949 - 0.989)	**0.002**
Lumbar puncture pressure	154	1.001 (0.999 - 1.004)	0.395		
Periventricular edema	154				
0	71	Reference			
1	83	1.379 (0.832 - 2.288)	0.213		
Ventricular enlargement	154				
1	75	Reference			
0	79	0.855 (0.521 - 1.403)	0.536		
Extra-cranial metastasis	154				
1	86	Reference		Reference	
0	68	0.458 (0.271 - 0.775)	**0.004**	0.585 (0.337 - 1.017)	0.050
LDH	154	1.002 (0.998 - 1.005)	0.300		
GLU	154	0.506 (0.383 - 0.669)	**< 0.001**	0.619 (0.454 - 0.844)	**0.002**
CL	154	0.936 (0.889 - 0.985)	**0.010**	0.999 (0.943 - 1.059)	0.985
PRO	154				
high	38	Reference		Reference	
medium	78	0.403 (0.224 - 0.728)	**0.003**	0.460 (0.247 - 0.859)	**0.015**
low	38	0.128 (0.055 - 0.298)	**< 0.001**	0.278 (0.110 - 0.706)	**0.007**
MR nodular enhancement	154				
1	74	Reference		Reference	
0	80	0.366 (0.218 - 0.615)	**< 0.001**	0.347 (0.201 - 0.600)	**< 0.001**
MR linear enhancement	154				
1	81	Reference		Reference	
0	73	1.544 (0.936 - 2.549)	0.089	1.346 (0.793 - 2.282)	0.271

Bold values indicate statistical significance.

Multivariate analysis confirmed that independent predictors of favorable survival included higher KPS (HR = 0.966, *p* = 0.001), absence of extracranial metastasis (HR = 0.582, *p* = 0.046), normal CSF glucose (HR = 0.601, *p* = 0.001), lower CSF protein levels (HR = 1.000, *p* = 0.042), and absence of MR nodular enhancement (HR = 0.383, *p* < 0.001).

A prognostic nomogram was subsequently constructed to predict 1-year survival probability (**Figure 6a**). Each independent variable contributed proportionally to the total risk score, enabling individualized OS estimation. Time-dependent ROC analysis for 1-year OS produced an AUC of 0.894, reflecting unadjusted discriminatory performance (**Figure 6b**). To account for internal overfitting, 1000-bootstrap resampling was implemented for formal model validation, yielding an original C-index of 0.792 and an optimism-corrected C-index of 0.777 (**Supplementary Table S5**). The apparent AUC value is relatively optimistic, while the bootstrap-corrected C-index offers a more conservative, reliable assessment of the model’s true predictive power. At the optimal Youden-index cutoff of -0.6914 for 1-year mortality risk stratification, the nomogram yielded a sensitivity of 0.647, perfect specificity of 1.000, positive predictive value (PPV) of 1.000, and negative predictive value (NPV) of 0.740; all diagnostic metrics are comprehensively summarized in **Supplementary Table S4**. Calibration analysis revealed excellent agreement between predicted and observed outcomes, supporting the reliability of the prognostic model (**Figure 6c**). Decision curve analysis (DCA) further assessed the clinical net benefit of the nomogram (**Figure 6d**). The nomogram curve maintained positive net benefit across threshold probabilities from 0 to 0.75, and its net clinical benefit was consistently superior to the strategies of universal intervention and no intervention within this clinically meaningful risk range.

**Figure 6:**
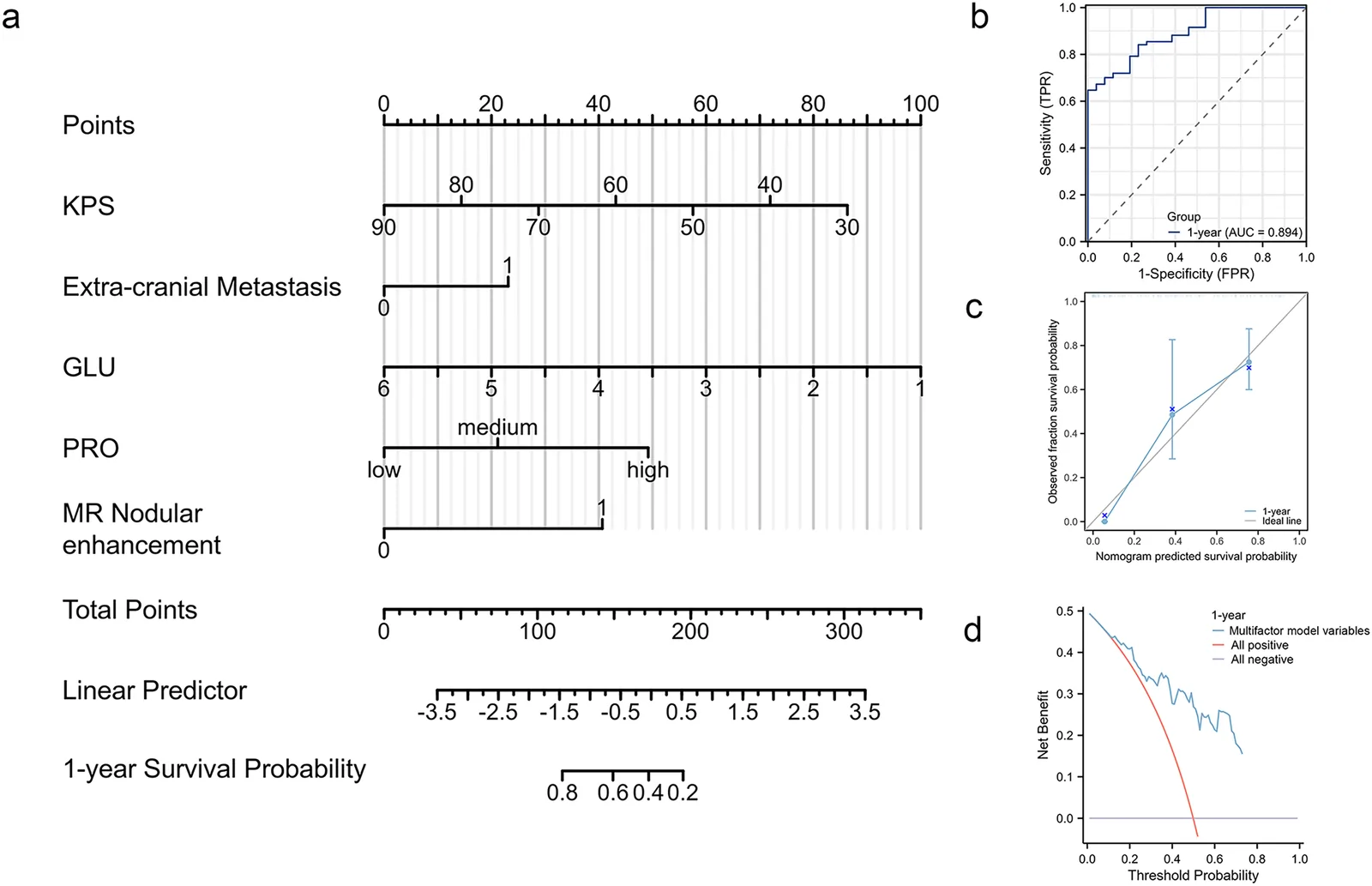
Prognostic model development and validation. **(a)** A prognostic nomogram constructed to predict 1-year survival probability, incorporating independent predictors: KPS, extracranial metastasis, CSF glucose, CSF protein, and MR nodular enhancement. **(b)** ROC curve demonstrating the robust discriminative ability of the prognostic model for predicting 1-year survival (AUC = 0.894). **(c)** Calibration plot showing excellent agreement between the nomogram-predicted probability of 1-year survival and the observed outcome. **(d)** DCA comparing the net clinical benefit of the nomogram against two default strategies across the full range of risk thresholds.

## 4 Discussion

This study provided a comprehensive evaluation of the clinical outcomes of Ommaya-based intrathecal pemetrexed and identified key prognostic determinants. The large sample size (n=154) strengthens these findings, providing a reliable framework for the clinical management of LCLM. Female sex, higher KPS, lower CSF protein/ICP, and linear MR enhancement were all associated with better survival. Together, these findings provide a foundation for developing individualized treatment strategies.

Ommaya-based IT administration bypasses the BBB and delivers chemotherapy directly into the CSF. This approach distributes drugs more effectively along CSF flow pathways, resulting in higher local concentrations and more uniform cytotoxic exposure than LP-based delivery (29, 30). Prior meta-analyses have demonstrated that intrathecal chemotherapy administered via an Ommaya reservoir yields a superior safety profile compared with repeated LP-based delivery (31). In our study, the median OS was 331 days, in concordance with prior reports (10, 12, 32, 33). Notably, female patients exhibited significantly better prognoses than male patients. We hypothesize this crude survival difference may reflect estrogen-mediated modulation of the leptomeningeal tumor microenvironment, yet this mechanistic pathway lacks dedicated translational or preclinical validation in LCLM cohorts; this interpretation is exploratory conjecture rather than confirmed causal evidence and requires targeted basic research for verification. Female gender showed a crude protective association with prolonged overall survival in univariate Cox regression (HR = 0.546, 95%CI 0.329-0.909, *P* = 0.020). This association was fully attenuated after adjustment for all clinically relevant confounders in multivariate analysis (HR = 0.784, 95%CI 0.444-1.382, *P* = 0.400). The univariate survival difference between sexes was driven by imbalanced baseline disease and cerebrospinal fluid characteristics rather than an independent sex-specific biological effect; gender was not validated as an independent predictor of mortality in the fully adjusted model. Higher KPS was strongly associated with improved prognosis, emphasizing that pretreatment physical condition was a key predictor of therapeutic response.

CSF cytology was positive in 87.7% of patients before treatment, confirming substantial leptomeningeal tumor burden at baseline. In our prognostic analysis, lower CSF protein levels and normal CSF glucose were both associated with significantly improved survival. These findings may reflect multiple interrelated mechanisms. On one hand, lower CSF protein could indicate preserved BBB integrity and reduced protein exudation, which may allow intrathecal chemotherapeutic agents to maintain higher and more sustained local concentrations, thereby enhancing therapeutic efficacy. Conversely, elevated CSF protein-often accompanied by barrier disruption-may lead to uneven drug distribution and diminished treatment response (34–36). On the other hand, lower CSF protein levels may simply reflect a lower tumor burden within the leptomeningeal space, as malignant cells and tumor-derived secretory products are major contributors to CSF protein elevation. Thus, CSF protein may serve as a surrogate marker of disease volume rather than a direct indicator of barrier integrity. This interpretation is supported by the observed correlations between CSF protein and other markers of disease activity, including CSF cytology positivity and MRI enhancement patterns. Collectively, these findings highlight the potential of CSF biochemical parameters as readily available prognostic biomarkers in LCLM. Moreover, the prognostic value of CSF glucose appeared related to metabolic features of the tumor microenvironment. Normal glucose levels were suggestive of intact metabolic status or reduced tumor consumption, supporting better treatment tolerance and suppressing tumor invasiveness (18–20). Abnormal glucose metabolism altered CSF composition and diminished chemotherapy sensitivity, consistent with poorer outcomes in hypoglycorrhachia (6, 25). These findings highlight the potential of CSF biochemical markers as a dynamic monitoring tool. Early shifts in protein and glucose levels could precede radiographic changes and provide an opportunity for early clinical intervention. Future studies incorporating circulating tumor DNA (ctDNA) profiling may further delineate the mechanisms of treatment resistance (37). Lower cerebrospinal fluid chloride levels correlated with worse overall survival in univariate screening (HR = 0.936, 95%CI 0.889-0.985, *P* = 0.010). However, this statistical association disappeared completely after multivariate adjustment (HR = 0.999, 95%CI 0.943-1.059, *P* = 0.985). Abnormal CSF chloride concentrations represent only a secondary electrolyte disturbance secondary to elevated intracranial pressure and disrupted blood-CSF barrier function, with no independent predictive value for mortality risk in leptomeningeal metastasis patients.

MRI is a critical tool in the diagnosis and prognostic assessment of LM. In our study, distinct MRI enhancement patterns carried significantly different prognostic implications. Nodular enhancement was associated with markedly shorter OS, higher ICP, and limited neurological recovery. Although elevated ICP was also frequently observed in patients with linear enhancement, their clinical outcomes were significantly better, suggesting that enhancement morphology conveys more prognostic information than ICP alone. Several mechanisms may underlie this disparity. Nodular lesions, characterized by dense tumor infiltration, prominent neovascularization, and a compact, mass-like architecture, can obstruct CSF circulation and create a physical barrier that restricts direct penetration of IT chemotherapeutic agents into the tumor tissue itself. These features, together with an immunosuppressive microenvironment, collectively limit drug delivery and therapeutic efficacy. In contrast, linear enhancement represents diffuse leptomeningeal infiltration with less severe CSF flow obstruction. The more homogeneous tumor distribution along the meningeal surface permits more consistent IT drug dispersion, potentially improving local cytotoxicity and clinical outcomes (38, 39). These findings underscore that MRI enhancement patterns—particularly the presence of nodular lesions—may serve as a valuable imaging biomarker for risk stratification and may guide more intensive or combined therapeutic strategies in high-risk patients. Of note, MRI was negative for meningeal involvement in approximately 24% of LM patients, highlighting the need for clinical vigilance when cranial nerve–related symptoms such as seizure, headache, nausea, vomiting, hearing loss, or visual disturbances occur. Hence, CSF analysis via LP remains essential in such cases to prevent delayed or misdiagnosis. Collectively, these findings supported integrating MRI patterns and CSF evaluation to guide individualized therapy. Patients with MR nodular enhancement may benefit from IT chemotherapy combined with targeted agents or immunotherapy to address drug-delivery limitations. Future studies linking MRI phenotypes with molecular and CSF biomarkers will be crucial to elucidate the mechanisms underlying enhancement patterns and optimize LM therapy (40, 41).

We examined the relationship between ICP, MRI patterns, and survival. Among our cohort, 61.0% of patients exhibited increased ICP. Although VP shunt surgery could provide durable relief of intracranial hypertension, ICP itself showed no significant association with survival in the overall cohort, suggesting that the ICP value did not fully reflect the pathological progression of LM. Subgroup analysis showed that elevated ICP predicted poorer survival in patients with KPS ≥ 70, but not in those with KPS ≤ 50. This likely reflects the greater compensatory capacity in high-KPS patients, where ICP elevation more directly signals tumor-related CSF obstruction (42–44). No published animal models reliably recapitulate progressive intracranial hypertension in human LCLM, and no preclinical research has formally validated a quantitative ICP-survival prognostic gradient comparable to our clinical observation. This ICP-related prognostic association identified in our retrospective cohort lacks direct animal experimental verification, which represents an important limitation of the current translational evidence base. Patients with a high KPS and low ICP were indicative of earlier stages of LM, and consequently showed better prognosis and stronger responses to IT. Conversely, a poor prognosis was observed in patients with low KPS and poor nutritional status, and ICP no longer had any prognostic relevance in this subgroup. An important innovation of our study is the development of a clinically informative four-tier grading system integrating KPS and ICP. By classifying patients into four distinct categories, this system captures the combined effects of functional status and CSF dynamics—two factors that are individually prognostic but rarely evaluated together in LM. The stepwise increase in HR across Grades 1 to 4 underscores its strong stratification capacity within limited patient subgroups, suggesting that the interplay between neurological performance and intracranial hypertension is a key determinant of survival. This integrative grading framework may therefore provide clinicians with a practical tool for risk assessment, treatment decision-making, and early identification of patients who are unlikely to benefit from intensive intrathecal therapy. Notably, this four-tier stratification tool has restricted clinical generalizability, originating from our clinical finding that isolated ICP measurement lacks sufficient prognostic value. It was exclusively constructed for patients with obvious neurological differentiation (KPS ≥ 70 or KPS ≤ 50), while moderate-KPS patients (51-69, n = 52) fall outside its applicable scope and cannot be stratified via this system. Further prospective validation could establish this model as a standardized metric for future LM management and clinical trial design. Importantly, our findings also suggested a potential therapeutic implication. In patients with early-stage disease who presented with elevated ICP, timely VP shunting not only alleviated intracranial hypertension and improved functional status but was also associated with extended OS, highlighting the value of early intervention in appropriately selected patients. For those with KPS < 50, although ICP level itself did not influence survival, VP shunting still offered meaningful clinical benefit by improving neurological function, relieving distressing symptoms, and reducing caregiver burden. These observations underscore the complementary role of VP shunting in LCLM management across different functional states.

In addition to its prognostic implications, our study also evaluated the procedural safety of Ommaya reservoir implantation combined with intrathecal chemotherapy in patients with LCLM. During the intrathecal chemotherapy phase, the major concern was seizure activity, particularly status epilepticus, which represents a serious adverse event and an established risk factor for poor neurological outcomes in LCLM. Such episodes may lead to severe or even irreversible CNS injury, underscoring the need for preventive strategies. Based on these observations, we propose a technical refinement aimed at reducing seizure risk associated with intrathecal pemetrexed administration. Specifically, positioning the Ommaya catheter tip within the lateral ventricle—while avoiding deeper advancement toward or into the third ventricle—may facilitate broader drug dispersion during intrathecal infusion. This approach reduces focal drug accumulation and minimizes direct irritation of the ependymal surface, thereby potentially lowering neurotoxic complications. However, our proposed refinement (restricting catheter tip to lateral ventricle to reduce seizure risk) is derived solely from single-center cumulative retrospective clinical experience. No dedicated prospective anatomical imaging, compartmental CSF pharmacokinetic, or head-to-head comparative clinical trials have been published to formally validate the neuroprotective benefit of this positioning strategy. This technical tip lacks high-level prospective evidence and requires independent external validation before generalized clinical adoption. The anatomical and pharmacokinetic rationale of this modification suggests that optimized catheter placement could enhance both the safety and tolerability of intrathecal chemotherapy, offering a practical reference for improving clinical implementation.

The prognostic model developed in this study integrated multiple clinical, radiographic, and biochemical parameters to improve survival prediction in LCLM. Female sex, higher KPS, lower CSF protein, higher CSF glucose, normal ICP, absence of extracranial metastasis, and linear MRI enhancement were all independent predictors of improved survival. These variables support stratified risk assessment and may assist clinicians in refining IT chemotherapy schedules or considering combination treatments. A notable strength of this study is its comprehensive diagnostic framework, which did not rely solely on positive CSF cytology, thereby capturing a broader and more clinically representative LCLM population (25).

Despite its strengths, this study had several limitations. As a single-center retrospective analysis, unavoidable selection bias may influence the findings. However, the relatively large sample size helped mitigate this concern. Additionally, confounding variables such as comorbidities and concomitant treatments could not be fully controlled. Radiotherapy was administered in some patients either before or after IT chemotherapy based on individual clinical indications; however, detailed radiotherapy data were not systematically available for analysis, and its potential confounding effect represents a limitation of this study. Finally, we did not perform a comparative analysis of different IT agents, such as pemetrexed versus methotrexate, which remains a potential area for future investigation. Together, these findings advance our understanding of LCLM biology and treatment responsiveness. As therapeutic options continue to evolve, integrating clinical, radiographic, and molecular biomarkers will be essential for optimizing patient selection and guiding individualized therapy.

## 5 Conclusion

In conclusion, this retrospective cohort summarizes the clinical outcomes of patients with LCLM who received Ommaya reservoir-based intrathecal pemetrexed (with or without concurrent ventriculoperitoneal shunt). Multivariate Cox regression identified independent prognostic factors including baseline KPS, extracranial metastasis status, meningeal MRI enhancement patterns, and CSF glucose/protein levels. We developed two exploratory risk-assessment tools based on these independent predictors: a four-tier KPS–ICP grouping scheme for simple clinical stratification, and a Cox nomogram to predict 1-year overall survival probability. Internal bootstrap validation demonstrated acceptable discriminative and calibration performance of the nomogram within the present cohort. The present observational data provide a reference framework for individualized prognostic evaluation and multidisciplinary management of LCLM patients treated with intraventricular chemotherapy. External prospective multi-cohort validation is required to confirm the generalizability of our risk stratification system and predictive model, and further research is needed to optimize intrathecal treatment regimens for this patient population.

## Data Availability

The raw data supporting the conclusions of this article will be made available by the authors, without undue reservation.
