# Supplementary material for: Clinical outcomes and prognostic factors of Ommaya reservoir-based intrathecal chemotherapy in lung cancer leptomeningeal metastasis: a retrospective cohort study
Source: Front Oncol. 2026 Sep 28;16:1915711. doi: 10.3389/fonc.2026.1915711 (PMC13654709; doi:10.3389/fonc.2026.1915711)
Supplement: Supplementary file 1 [file Table1.docx]

Table S1. Baseline clinical comparisons between patients with and without VP shunt implantation.

| Characteristics | VP Shunt | No VP Shunt | *P* value |
| --- | --- | --- | --- |
| n | 90 | 64 |  |
| Age, median (IQR) | 58 (51, 63) | 61 (57, 68) | 0.001 |
| Gender, n (%) |  |  | 0.204 |
| Female | 57 (37%) | 34 (22.1%) |  |
| Male | 33 (21.4%) | 30 (19.5%) |  |
| KPS, median (IQR) | 60 (50, 70) | 60 (50, 62.5) | 0.262 |
| ICP, median (IQR) | 270 (220, 310) | 155 (130, 180) | < 0.001 |
| Ventricular enlargement, n (%) |  |  | 0.300 |
| 1 | 47 (30.5%) | 28 (18.2%) |  |
| 0 | 43 (27.9%) | 36 (23.4%) |  |
| Periventricular edema, n (%) |  |  | < 0.001 |
| 1 | 60 (39%) | 23 (14.9%) |  |
| 0 | 30 (19.5%) | 41 (26.6%) |  |
| PRO, median (IQR) | 616.5 (453, 813) | 647 (452.5, 830.5) | 0.837 |
| GLU, median (IQR) | 2.225 (1.3625, 3.1225) | 2.525 (1.4375, 3.32) | 0.507 |
| LDH, median (IQR) | 42.5 (22, 59) | 32.5 (17.75, 58.5) | 0.120 |
| Extracranial metastasis, n (%) |  |  | 0.807 |
| 1 | 51 (33.1%) | 35 (22.7%) |  |
| 0 | 39 (25.3%) | 29 (18.8%) |  |
| OS, median (IQR) | 325.5 (268, 442.25) | 352.5 (266, 437) | 0.680 |

Table S2 Survival outcomes across the four-tier KPS-ICP prognostic grading system.

| **Grading definition** | **N** | **Events** | **Median OS, days (95% CI)** | **HR (95% CI) vs Grade 1** | **Cox *P*-value** |
| --- | --- | --- | --- | --- | --- |
| Grade1: KPS ≥ 70 + ICP < 200 | 13 | 1 | Not reached (266–∞) | Reference | – |
| Grade2: KPS ≥ 70 + ICP ≥ 200 | 30 | 11 | 390 (298–764) | 3.401 (1.406–3.900) | <0.05 |
| Grade3: KPS ≤ 50 + ICP ≥ 200 | 31 | 17 | 380 (336–718) | 3.809 (2.347–4.670) | <0.05 |
| Grade4: KPS ≤ 50 + ICP < 200 | 26 | 16 | 326 (317–787) | 4.228 (3.578–7.330) | <0.05 |

Table S3. Bonferroni-corrected pairwise log-rank P values for overall survival comparisons between the four KPS-ICP prognostic grades.

| **Comparison** | **Corrected P-value** | **Raw unadjusted P** |
| --- | --- | --- |
| Grade1 vs Grade2 | 1.0000 | 0.1820 |
| Grade1 vs Grade3 | 0.3910 | 0.0652 |
| Grade1 vs Grade4 | 0.1593 | 0.0266 |
| Grade2 vs Grade3 | 1.0000 | 0.2005 |
| Grade2 vs Grade4 | 0.2906 | 0.0484 |
| Grade3 vs Grade4 | 1.0000 | 0.6651 |

Table S4. Diagnostic performance metrics of the nomogram for predicting 1-year overall survival at the optimal Youden-index risk cutoff.

| **Parameter** | **Value** |
| --- | --- |
| Predicted time point | 365 days (1-year OS) |
| Optimal risk threshold (cutoff) | -0.691426313 |
| True positive (TP) | 0.647058171 |
| False positive (FP) | 0 |
| Positive predictive value (PPV) | 1.000 |
| Negative predictive value (NPV) | 0.74005062 |
| Sensitivity | 0.647058171 |
| Specificity | 1.000 |

Table S5. Internal validation indices of the prognostic nomogram

| **Calibration parameter** | **Value** |
| --- | --- |
| Bootstrap calibration slope (1-year OS) | 1.049 |
| Bootstrap calibration intercept (1-year OS) | −0.049 |
| Original C-index | 0.792 |
| Bootstrap-corrected C-index | 0.777 |
